# Toxicological Characteristics of Bacterial Nanocellulose in an In Vivo Experiment—Part 2: Immunological Endpoints, Influence on the Intestinal Barrier and Microbiome

**DOI:** 10.3390/nano14201678

**Published:** 2024-10-19

**Authors:** Vladimir A. Shipelin, Ekaterina A. Skiba, Vera V. Budaeva, Antonina A. Shumakova, Eleonora N. Trushina, Oksana K. Mustafina, Yuliya M. Markova, Nikolay A. Riger, Ivan V. Gmoshinski, Svetlana A. Sheveleva, Sergey A. Khotimchenko, Dmitry B. Nikityuk

**Affiliations:** 1 Federal Research Centre of Nutrition and Biotechnology, 109240 Moscow, Russia; antonina_sh@list.ru (A.A.S.); trushina@ion.ru (E.N.T.); mustafina@ion.ru (O.K.M.); yulia.markova.ion@gmail.com (Y.M.M.); n_rieger@icloud.com (N.A.R.); gmosh@ion.ru (I.V.G.); sheveleva@ion.ru (S.A.S.); hotimchenko@ion.ru (S.A.K.);; 2 Institute for Problems of Chemical and Energetic Technologies of the Siberian Branch of the Russian Academy of Sciences, 659322 Biysk, Russia; eas08988@mail.ru (E.A.S.); budaeva@ipcet.ru (V.V.B.); 3 Department of Food Hygiene and Toxicology, Institute of Vocational Education, I.M. Sechenov First Moscow State Medical University, 119435 Moscow, Russia; 4 Department of Operative Surgery and Topographic Anatomy, I.M. Sechenov First Moscow State Medical University, 119435 Moscow, Russia; 5 Department of Ecology and Food Safety, Institute of Ecology, Peoples’ Friendship University of Russia Named After Patrice Lumumba, 117198 Moscow, Russia

**Keywords:** bacterial cellulose, nanofibers, toxicity, lymphocytes, cytokines, intestinal barrier, intestinal microbiome, anaphylaxis, rats

## Abstract

Bacterial nanocellulose (BNC) is considered a promising alternative to microcrystalline cellulose, as well as an ingredient in low-calorie dietary products. However, the risks of BNC when consumed with food are not well characterized. The aim of this study is to investigate the impact of BNC on immune function, the intestinal microbiome, intestinal barrier integrity, and allergic sensitization in subacute experiments on rats. Male Wistar rats received BNC with a diet for eight weeks in a dose range of 1–100 mg/kg of body weight. The measurements of serum levels of cytokines, adipokines, iFABP2, indicators of cellular immunity, composition of the intestinal microbiome, and a histological study of the ileal mucosa were performed. In a separate four-week experiment on a model of systemic anaphylaxis to food antigen, BNC at a dose of 100 mg/kg of body weight did not increase the severity of the reaction or change the response of IgG antibodies. Based on dose–response effects on immune function, the non-observed adverse effect level for BNC was less than 100 mg/kg of body weight per day. The effects of BNC on the gut microbiome and the intestinal mucosal barrier were not dose-dependent. Data on the possible presence of prebiotic effects in BNC have been obtained.

## 1. Introduction

Bacterial nanocellulose (BNC) is a promising product of modern bio- and nanotechnology, which has favorable physicochemical characteristics, such as high adsorption capacity and the ability to form stable hydrogels [1,2]. In this regard, BNC has been proposed for use in food as a potential food additive with the technological functions of a thickener, emulsifier, stabilizer, and gelling agent [3] instead of the traditionally used microcrystalline cellulose from wood (E460i) as well as a source of insoluble dietary fiber in the composition of functional and dietary low-calorie food products.

Products such as “Nata de Coco” and “Kombucha” have a long tradition of food use in Southeast Asian cuisine for the preparation of desserts and beverages. While “Nata de Coco” is a BNC-containing food, “Kombucha” is a clear drink that does not contain BNC despite being produced during the fermentation process [4,5]. BNC, as a food and dietary supplement, has spread worldwide from Southeast Asia since the twentieth century and, according to recent reviews, currently displays a significant upward trend in human consumption [6,7]. However, information on the safety of BNC produced on an industrial scale using microbial fermentation with symbiotic cultures is limited. Due to this, BNC is currently not listed as an approved food additive in the European Union [8], nor has GRAS (Generally Recognized as Safe) status in the United States [9]. In Russia, BNC is not included in the list of food additives approved for use. Despite this, BNC is already produced on an industrial scale in Japan and the Philippines, including for export to the USA and European countries for food use [3].

In research conducted in Russia by researchers from the Institute for Problems of Chemical and Energetic Technologies, a technology for the production of BNC was developed by fermentation of various types of plant biomass with a symbiotic culture of *Medusomyces gisevii* [10]. BNC obtained in this way is characterized by the high content of cellulose allomorph Iα (CIα) in the range of 94–100% and degree of crystallinity (CI) in the range of 88–93%, which determines the high strength of its fibers, water-holding, and gel-forming ability, and makes it an attractive object for use as a food ingredient. However, in order to register this type of BNC as a new food product, it is necessary to conduct comprehensive toxicological and hygienic studies confirming its safety.

Our previous work [11] presented data on the effect of BNC produced by *Medusomyces gisevii* strain Sa-12 on behavioral response, integral and biochemical endpoints, the safety of vitamins A and E, the expression of antioxidant defense and apoptosis proteins, and the morphology of the liver and kidneys of male rats in an 8-week subacute experiment. This study aimed to further toxicological and hygienic characterization of BNC from *Medusomyces gisevii* in experiments on rats with an assessment of the effect on immune function, intestinal microbiome, morphofunctional state of the intestinal mucosal barrier, and evaluation of allergenic action.

## 2. Materials and Methods

### 2.1. Samples of Bacterial Nanocellulose

BNC was obtained at the Federal State Budgetary Institution of Science Institute for Problems of Chemical and Energetic Technologies (Siberian Branch of the Russian Academy of Sciences, Biysk, Altai Krai, Russia) by the fermentation with a symbiotic culture of *Medusomyces gisevii* strain Sa-12, in a semi-synthetic glucose medium according to the method presented earlier [10]. According to the electron microscopic characterization presented in detail in [11], the ultrastructure of the BNC sample was distinguished by the presence of fibers more than 10 μm in length and about 50 nm in diameter, formed by parallel bundles of nanofibrils with a diameter of 5–10 nm or less, some of which were separated from the main fiber and were present in the form of short fragments with a length of 50 to 200 nm. Thus, the presented BNC sample can be defined as an industrial nanomaterial represented by nanofibers according to ISO/TS 80004-2 [12].

Studies conducted at the Testing Laboratory Center of the Federal Research Centre of Nutrition and Biotechnology showed that the obtained sample of BNC (in the form of a hydrogel with a dry matter content of 1.4%) corresponds to safety indicators established in Commission Regulation (EU) No 231/2012 of 9 March 2012 in application to MCC of the plant origin food additive (E460(i)), in terms of toxic element (arsenic, cadmium, lead, mercury) content and is also characterized by the absence of twenty-four mycotoxins (presented in detail in [11]).

### 2.2. Experimental Design

Experiments were carried out on male rats of the outbred line Wistar, obtained from the Stolbovaya nursery of the Federal State Budgetary Institution Scientific Center for Biology and Medical Sciences of the Federal Medical and Biological Agency of Russia. Procedures for animal housing and experiments were in accordance with international guidelines for good laboratory practice [13]. The experimental design was approved by the Ethics Committee of the Federal Research Center of Nutrition and Biotechnology, protocol No. 7, dated 14 October 2022.

In the first experiment, 48 rats aged six weeks with an initial body weight (b.w.) of 123 ± 3 g (hereinafter M ± s.e.m.) were used and were divided into four groups of 12 individuals that did not differ in the initial average b.w. (*p* > 0.1, ANOVA test). A detailed description of the experimental design was given in a previous publication [11]. Rats received BNC for eight weeks at calculated doses of 0 (group 1, control), 1.0 (2nd group), 10.0 (3rd group), and 100 mg/kg b.w. (4th group) as part of a balanced semi-synthetic diet composed in accordance with AIN-93M. The actual dose of BNC consumed was determined daily by weighing the remaining feed, and if necessary, the amount added to the diet was adjusted. As a result, the given doses of BNC were maintained stably throughout the entire feeding period [11].

Animals were removed from the experiment on the 57th day after a 16 h fast by decapitation under ether anesthesia. Under aseptic conditions, blood was collected immediately after decapitation for the analysis of immunological and hematological parameters, the ileum for morphological studies, and the cecum for analysis of the composition of the intestinal microbiome.

In the second experiment, the effect of BNC on the severity of an allergic reaction in rats was studied in a model of systemic anaphylaxis (SA) to a model food antigen–chicken egg ovalbumin (OVA). We used two groups of 24 rats, eight weeks old, with an average initial body weight of 236 ± 15 g (group 1a, control) and 241 ± 20 g (group 2a, experimental). The difference in body weights was not significant, *p* > 0.1, ANOVA test. Rats of both groups were sensitized intraperitoneally with 5-fold recrystallized OVA at a dose of 100 μg along with 10 mg of aluminum hydroxide used as an adjuvant on the 1st, 3rd, and 5th days of the experiment. On the 21st day, an additional dose of 10 μg of OVA was administered under the same conditions. The control group of animals received a balanced semi-synthetic diet according to AIN-93M, and the experimental group received the same diet with the addition of BNC at a calculated dose of 100 mg/kg b.w. per day. The condition of the animals was observed, including a visual assessment of the nature of the behavior, activity, type of coat, and mucous membranes; b.w. was determined once a week on electronic balance with an error of ±1 g. The total duration of feeding was four weeks. On the 29th day, SA was initiated by intravenous administration of OVA at a dose of 6 mg/kg b.w. in sterile pyrogen-free 0.15 M NaCl grade “for injection”. The OVA solution for administration was sterilized by passing through a filter with a pore diameter of 0.22 μm. Immediately before injection, blood was collected from the tail vein to determine the level of specific IgG antibodies to OVA. The progress of SA was observed within 24 h after its introduction, and the severity of the reaction was assessed in points according to the scale: (0) no reaction; + (1) chills, shortness of breath; ++ (2) weakness, ataxia, cyanosis of the limbs; +++ (3) convulsions, paralysis; and ++++ (4) lethal outcome. Surviving animals were removed from the experiment by inhalation of a lethal dose of CO_2_.

### 2.3. Hematological and Immunological Methods of Study

Hematological parameters of erythrocytes, leukocytes, and platelets of peripheral blood were determined on a Coulter ACTTM 5 diff OV hematological analyzer (Beckman Coulter, Indianapolis IN, USA) with a set of reagents produced by Beckman Coulter, France, and according to the manufacturer’s methods.

The expression of the cellular antigens CD45R, CD3, CD4, CD8a, and CD161 on lymphocytes (Ly) of the peripheral blood of rats was determined by direct immunofluorescent staining of whole blood cells using a panel of monoclonal antibodies conjugated with fluorescent dyes APC, FITC, and PE (manufactured by Miltenyi Biotec GmbH, Gladbach, Germany). Lysis/fixation and staining of cells were performed according to the manufacturer’s instructions. Lymphocyte populations were isolated by gating on parameters such as small angle (FS) and side (SS) of light scattering. Next was the gating of CD3+ lymphocyte populations via fluorescence channels FL1 and SS Lin. Results were recorded on a two-parameter histogram distribution of CD3+ (from gate B) using monoclonal antibodies against CD4 and CD8, detected in fluorescence channels FL5 and FL4, respectively. Similarly, in a separate test, the expressions of CD45RA and CD161a were determined. The content of CD45R+ cells (B-Ly), CD3+ (T-Ly), and CD161+ (NK-cells) was expressed as % of the total number of analyzed cells; the content of CD3+ CD4+ (T-helper cells) and CD3+ CD8+ (T-cytotoxic Ly) was expressed as % of the total number of CD3+ cells. The dimensionless immunoregulatory index (IRI) was calculated as a ratio of CD4+ to CD8+ Ly number. The measurements were performed on a flow cytometer FC-500 manufactured by Beckman Coulter (USA) according to the manufacturer’s method.

Determination of blood serum levels (pg/mL) of cytokines IFN-g, IL-10, IL-12 (p70), IL-1a, IL-4, IL-5, IL-6, MIP-1a, TNF-a, IL-17A, and adipokines (ghrelin and leptin) were performed using a commercial Bio-Plex Pro™ Reagent Kit V supplemented with Bio-Plex Pro™ Rat 33-Plex Standards, Rat Cytokine IL-1a Set, Rat Cytokine IL-4 reagents Set, Rat Cytokine IL-5, Set, Rat Cytokine IL-6 Set, Rat Cytokine IFN-g Set, Rat Cytokine IL-10 Set, Rat Cytokine IL-12 (p70) Set, Rat Cytokine IL-17A Set, Rat Cytokine MIP-1a Set, Rat Cytokine TNF-a Set, Rat Diabetes Ghrelin Set, and Rat Diabetes Leptin Set from Bio-Rad Laboratories, Inc. (Bio-Rad, Hercules, CA, USA). During the analysis, blood plasma samples were diluted with sample dilution buffer to a final concentration of 1:4. The analysis procedure consisted of four stages. In the first stage, samples are mixed with capture microspheres specific to the studied factors and coated with antibodies specific to the target substance. In the second stage, microspheres with captured analytes are incubated with monoclonal biotinylated antibodies to form a complex: analyte-microsphere-detector antibodies. In the third stage, the formed complexes are incubated with a fluorescent label (SA–PE: streptavidin–phycoerythrin). In this case, the amount of attached fluorescent label is linearly related to the concentration of the analyte in given units. The results are recorded on a multiplex flow analyzer, Luminex 200 (Luminex Corporation, Austin, TX, USA), using xMAP technology with Luminex xPONENT Version 3.1 software (Luminex Corporation, USA). The content of cytokine IL-33, adipokine FGF-21, and the intestinal fatty acids binding protein iFABP2 was analyzed using enzyme-linked immunosorbent assay (ELISA) with kits from Cloud-Clone Corp. (Katy, TX, USA) according to the manufacturer’s instructions.

The concentration of specific antibodies to OVA of the IgG class in the blood serum of sensitized rats was determined by indirect solid-phase ELISA on polystyrene plates, adsorbed with 5-fold recrystallized OVA and using rabbit antibodies against rat IgG conjugated with peroxidase as secondary antibodies (Sigma-Aldrich, Jerusalem, Israel), and a ready-made chromogenic substrate hydrogen peroxide–TMB (tetramethylbenzidine) produced by Imtek LLC (Moscow, Russia). For all ELISAs, optical density was measured on a Multiscan FC photometer manufactured by Thermo Fisher Scientific (Vantaa, Finland) at a wavelength of 450 nm with background correction at 620 nm.

### 2.4. Methods of Microbiological Study

The composition of the intestinal microbiocenosis was studied by inoculating the contents of the cecum of rats in tenfold dilutions on specific nutrient media, as described in the manual [14]. The study of the microbial landscape of the contents of the cecum included the identification of general and hemolytic aerobes, general anaerobes, bifidobacteria, lactobacilli, common and citrate-assimilating enterobacteria, enterococci, Bacteroides, molds, and yeasts. The number of colony-forming units (CFU) of microorganisms was expressed as the decimal logarithm of CFU/g of cecal contents. The genera of microorganisms were identified by studying the morphology of colonies, microscopy of smears, and determination of differential diagnostic biochemical properties.

### 2.5. Histological Methods

Samples of the ileum of rats at a position of 5–10 cm above *Sphincter ileocaecalis* immediately after collection were cut along the junction with the mesentery, fixed in a 3.7% formaldehyde solution in 0.1 M sodium phosphate buffer pH 7.00 ± 0.05 for at least 3 days, dehydrated in alcohols of increasing concentration, soaked in xylene, and filled with homogenized Histomix paraffin medium. Paraffin sections 3–4 µm thick were prepared on a sled microtome, stained with hematoxylin-eosin, and examined in an AxioImager Zl microscope (Carl Zeiss, Oberkochen, Germany) equipped with a digital camera at a magnification of ×50 and ×100. Morphometry was performed using AxioVision Rel.4.8 software (Carl Zeiss) and an X/Y calibration slide with a scale division of 10 µm (manufactured by Mikromed, Moscow, Russia).

### 2.6. Statistical Processing of Experimental Data

When processing the statistical data, the sample mean M and the standard error mean (s.e.m.) were calculated. For data with a distribution that does not correspond to normal, the median, maximum, and minimum values, as well as the quartile interval, were determined. The correspondence of the distribution of values to the normal law was assessed using the Kolmogorov–Smirnov test. The significance of differences between groups was established using a one-way analysis of variance ANOVA and the nonparametric Mann–Whitney test as a post hoc test. The significance of differences in alternative indicators was established using Fisher’s exact U test and multivariate χ-squared test. Differences were accepted as significant at a level of *p* < 0.05. The calculations were performed using the statistical package of SPSS^®^ 23.0 and Microsoft Excel for Windows.

## 3. Results

### 3.1. Assessment of Hematological and Immunological Parameters in an 8-Week Experiment

**Figure 1:**
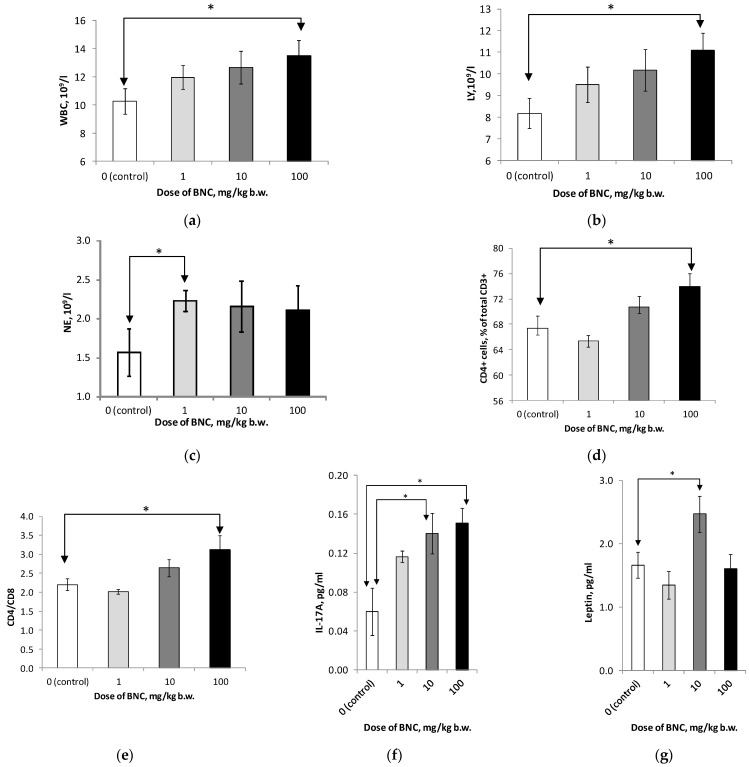
Immunological parameters of rats in an 8-week toxicological experiment. (**a**) Total leukocyte count in peripheral blood, 10^9^*L^−1^; (**b**) total lymphocyte count, 10^9^*L^−1^; (**c**) total number of neutrophils, 10^9^*L^−1^; (**d**) the number of CD4+ cells in % of the total number of CD3+ cells; (**e**) immunoregulatory index (the ratio of CD4+ to CD8+ cells); (**f**) serum IL-17A content, pg/ml; and (**g**) serum leptin content, pg/mL. Number of animals 10 (**a**–**e**) and 8 (**f**,**g**) per group. * The difference is significant for groups connected by an arrow, *p* < 0.05, Mann–Whitney test.

Consumption of BNC with diet by rats at the maximum dose (100 mg/kg b.w.) for eight weeks led to a significant (*p* < 0.05) increase in the number of leukocytes (Figure 1a) and Ly (Figure 1b) in the peripheral blood. Similar changes in these populations at the two lower doses of BNC were not statistically significant. At the same time, the number of blood neutrophils (Figure 1c) increased compared to the control approximately equally in all experimental groups but was statistically significant only in rats in group 2 (BNC, 1 mg/kg). As follows from the data in Figure 1d,e the relative proportion of CD4+ positive cells (T-helpers) in the total pool of T-Ly (CD3+) and the immunoregulatory index (CD4+/CD8+) increased with the dose of BNC consumed, and the difference with control was also significant in group 4 (BNC, 100 mg/kg b.w.).

Analysis of the cytokine profile of the blood serum of rats treated with BNC did not reveal dose-dependent and (or) statistically significant differences with the control group in the content of most of the studied cytokines and adipokines, such as IFN-g, IL-1a, IL-4, IL-5, IL- 6, IL-10, IL-12 (p70), IL-33, MIP-1a, TNF-a, FGF-21, and ghrelin (Supplementary Table S1). At the same time, noteworthy is the significant (for groups 3 and 4) increase in the level of IL-17A (Figure 1f), as well as the increase in leptin content in rats of group 3 (Figure 1g).

### 3.2. Effects on the Cecal Microbiome

**Figure 2:**
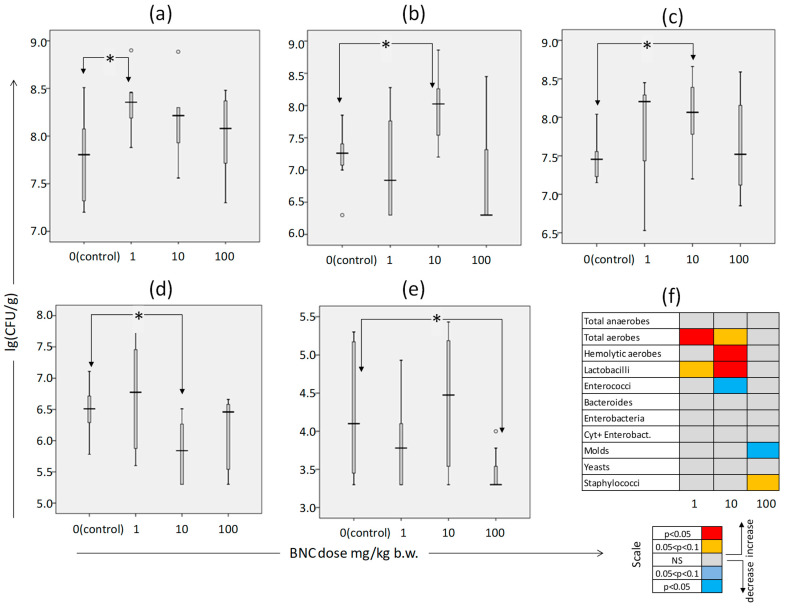
Indicators of the microbiome of the cecum of rats in an 8-week toxicological experiment. Content: (**a**) aerobic microorganisms; (**b**) hemolytic aerobes; (**c**) lactobacilli; (**d**) enterococci; and (**e**) molds. (**f**) A diagram (heat map) of the degree of discrepancy between the microbiome of the animals of the experimental groups and the control following the direction and statistical significance of the changes detected. The number of animals: 8 per group. Boxplot charts show the median value (horizontal bar), quartile range (box), range of change (bars), and outliers (circles). *—see Figure 1.

The effects of oral administration of BNC on some of the cecal microbiome populations studied did not have an unambiguous dependence on the dose of the nanomaterial. Thus, the following effects were observed: at a minimum dose of 1 mg/kg b.w. (group 2), a significant increase in the number of CFUs of total aerobes (Figure 2a), and at a dose of 10 mg/kg b.w. (group 3), an increase in the number of hemolytic aerobes (Figure 2b) and lactobacilli (Figure 2c), simultaneously with a decrease in enterococci (Figure 2d). In group 4, which received the highest dose of BNC, 100 mg/kg, the most noticeable was a decrease in the content of molds (Figure 2e). As presented in Figure 2f, the state of the remaining studied populations of microorganisms was stable and did not depend on the amount of BNC consumed. At the same time, the greatest number of changes in the state of the microbiome was noted in group 3, that is, with an intermediate (10 mg/kg b.w.) dose of BNC.

### 3.3. The Morphofunctional State of the Intestinal Wall

**Figure 3:**
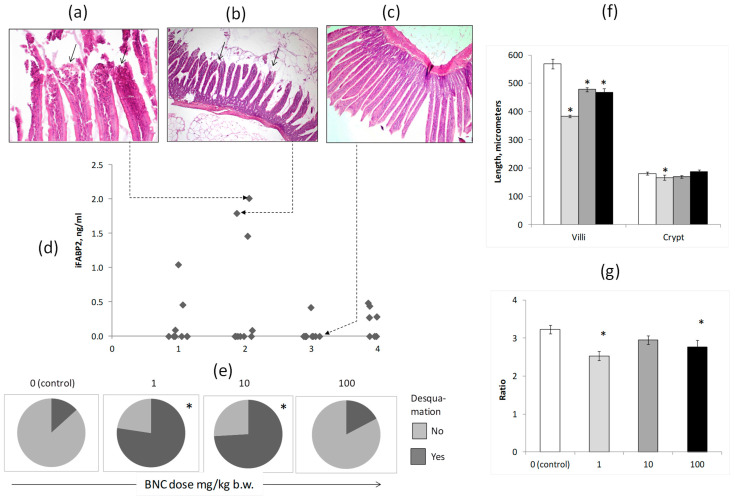
Indicators of the morphofunctional state of the ileum mucosa of rats in an 8-week toxicological experiment. Representative micrographs of the intestinal wall of rats from group 2 (**a**,**b**) and group 3 (**c**) in relation (arrows, dotted lines) to individual serum levels of fatty acid binding protein (iFABP2) (**d**); the proportion in % of the villi with a disturbed epithelial layer (**e**); average length of villi and depth of crypts by groups of animals, µm (**f**); and the ratio of the length of the villi to the depth of the crypts (**g**). Number of animals per group: 10 (**d**); 4 (**e**–**g**) with at least 45 pairs of villi and crypts examined on all slides from each group. *—a significant difference from group 1 (control), *p* < 0.05, Mann–Whitney test. Hematoxylin-eosin staining, ×100 magnification (**a**); ×50 (**b**,**c**).

The structure of the wall of the ileum of rats in the control group was characterized by the presence of developed villi of the mucous membrane, a well-defined layer of connective tissue and muscularis mucosa, moderate depth of the crypts (about 1/3 of the length of the villi), the presence of a large number of goblet cells producing intestinal mucus, such as in the villi and in the cryptal zone, and a minimal (less than 15%) number of villi with a damaged (desquamated) epithelial layer. At the same time, when BNC was consumed in a minimal dose (1 mg/kg b.w.), a significant number of villi appeared in the mucous membrane with signs of epithelial desquamation in the apical part (Figure 3a,b); at a dose of BNC 10 mg/kg b.w. (Figure 3c) the number of such villi was somewhat smaller. In some animals with severe signs of desquamation of the intestinal epithelium, increased levels of the fatty acid transport protein iFABP2 were noted in the blood serum, which, according to [15], is an informative biomarker of impaired macromolecular permeability of the intestinal barrier (Figure 3d). However, in general, the level of iFABP2 in the experimental groups of animals did not differ significantly from the control (*p* > 0.1, Mann–Whitney test), although there was a tendency towards its increase in group 2. The number of villi with signs of epithelial desquamation (Figure 3e) significantly increased in animals of groups 2 and 3; however, in group 4, which received BNC at the maximum dose, there was no difference from the control. As follows from the morphometric data, rats in group 2 (BNC 1 mg/kg b.w.) were characterized by the most pronounced (compared to the control) decrease in villi length, crypt depth (Figure 3f), and villus/crypt length ratio (Figure 3g). The changes in all these morphometric indicators were not dose-dependent.

### 3.4. Assessment of Allergenic Properties

**Figure 4:**
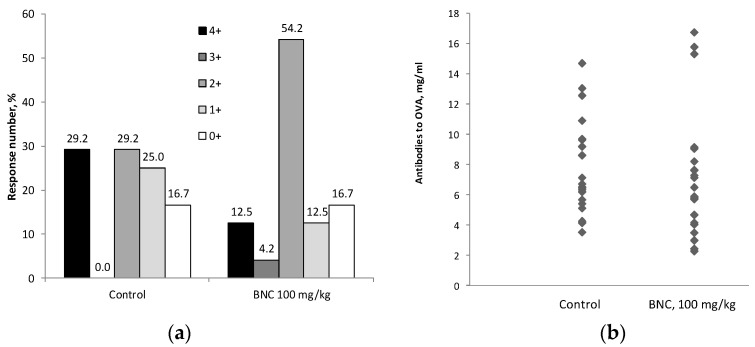
The results of reproducing the reaction of systemic anaphylaxis to OVA in rats of the control group and treated with BNC at a dose of 100 mg/kg b.w. (**a**) Distribution of animals of the two groups according to the severity of the anaphylaxis reaction, expressed in points. (**b**) Individual serum concentrations of specific IgG4 antibodies to OVA, mg/mL in two groups. The number of animals: 24 per group.

When evaluating the allergenic properties of BNC in a 28-day experiment, the rats in both groups—control and receiving BNC—steadily gained body weight, had a normal appearance, and no morbidity or mortality was observed during feeding with the diets. The b.w. of animals was 325.0 ± 4.5 g and 337.1 ± 6.1 g at the end of the experiment, respectively, and did not differ significantly (*p* > 0.1). Induction of SA reaction by intravenous administration of OVA led within 24 h to the death of 29.2% of rats in the control group and 12.5% in the experimental group (*p >* 0.1 Fisher’s exact U test). The distribution of animals according to the severity of anaphylactic shock represented in points (Figure 4a) showed a slight increase in the number of rats in the experimental group with a reaction of moderate severity (++) due to a decrease in the number of animals that gave a mild or, on the contrary, lethal anaphylaxis reaction. However, these distributions did not differ significantly (*p* > 0.1, multivariate c-squared test). As follows from Figure 4b, the level of specific IgG antibodies to OVA in rats of the control and experimental groups measured before administration of the challenge dose also differed insignificantly (*p* > 0.1, Mann–Whitney test). Thus, BNC, when consumed by rats with food at a dose of 100 mg/kg b.w. does not have a significant effect on sensitization to the model allergen and the severity of the anaphylaxis reaction, although there was an insignificant trend towards a decrease in the number of lethal reactions.

## 4. Discussion

Studies have shown that BNC in the dose range from 1 to 100 mg/kg b.w. has various local effects on the intestinal mucosa of rats and the state of the cecal microbiome. Previous studies using a limited number of indicators did not provide data indicating the presence of acute and subacute toxicity in BNC when administered orally in very high doses (from 2000 to 8000 mg/kg b.w.) [16,17,18]. In this regard, one should consider as a limitation of this study, firstly, the relatively narrow range of BNC doses studied in the subacute toxicological experiment, which in some cases made it difficult to compare the results with the data of the above studies. Secondly, the impossibility of evaluating the allergenic effect of BNC at higher doses due to the limited amount of available BNC and difficulties in its being administered into the diet in higher amounts.

The identified statistically significant dose-dependent effects of BNC, which can be taken into account when determining its maximum non-observed effect level (NOEL), include an increase in leukocytosis, the content of CD3+ and CD4+ Ly, the immunoregulatory index (CD4+/CD8+) together with the level of IL-17A, despite the fact that the effect on production of other cytokines and chemokines studied, including pro-inflammatory (IL-1a, IL-6, IL-12(p70), TNF-a, MIP-1a, INF-g), anti-inflammatory (IL-10), modulating metabolism (ghrelin, FGF-21), and increasing allergic sensitivity (IL-4, IL-5, IL-33), turns out to be insignificant. There is evidence that, when entering the body, various forms of nanocellulose can cause both local and systemic inflammatory reactions [19]. In light of this, it is worth noting that the biological significance of IL-17A, expressed by the Th17 subpopulation of helper CD4-positive lymphocytes, is to maintain the activity of a number of components of anti-infective and antitumor immunity with the development of concomitant inflammatory reactions [20] and can presumably be correlated with the overproduction of certain populations opportunistic intestinal microflora against the background of high BNC consumption. Qualitatively, the increase in IL-17A production is consistent with the increase in the number of neutrophils in animals treated with BNC, for which this cytokine is a stimulating factor [21]; however, this effect did not have a clear dependence on the dose of BNC, which makes it difficult to interpret this relationship unequivocally. Another possible arena of exposure to elevated levels of IL-17A may be the liver, in which the presence of cells producing this cytokine marks the presence of inflammatory and fibrotic processes [22]. It is known that one of the functions of IL-17A is the polarization of macrophages towards the M1 phenotype, which corresponds to the selective activation of this population under the influence of certain types of nanocellulose known from the literature [23]. In our previous study [11], as well as in works [24,25], the vacuolization of cells observed upon consumption of various types of nanocellulose in the periportal region of the liver parenchyma may presumably be the initial sign of the development of the inflammatory response caused by these processes.

The effect of BNC on the cytokine profile revealed in this study is relatively minor compared to previously obtained data for cellulose fibers of plant origin, which caused, among other things, an increase in the production of IL-5 and IL-10 in rats [26]. The absence of a pronounced effect of BNC on the levels of IL-4, responsible for the activation of B-lymphocytes and humoral immunity, and IL-5, which stimulates eosinophilia, is consistent with the lack of allergenic effect of BNC in the model of systemic anaphylaxis.

However, the signs of systemic inflammation observed at the maximum dose of BNC, monitored by hematological parameters and IL-17A production, require additional research from the perspective of the mechanisms of their development.

An increased serum level of leptin, detected at an intermediate (10 mg/kg b.w.) dose of BNC, is accompanied by the appearance of vacuolated hepatocytes in the liver of these animals. Numerous literature data indicate a connection between leptin levels, fatty acid metabolism regulated by the PPAR-g signaling pathway, liver lipodystrophy, on the one hand, and the expression of leptin and its receptor [27]. However, additional studies at the postgenomic level are required to establish the precise nature of this association in the case of BNC exposure. The increase in leptin levels in animals in group 3 is also qualitatively consistent with the presence of desquamation of the intestinal epithelium at this dose of BNC. There is evidence that, besides adipocytes, leptin is also produced by cells of the intestinal mucosa, and its increased level can mark the processes of damage to the cells of this organ [28,29].

The literature describes various changes in the intestinal microflora caused by animals’ consumption of plant cellulose in nanoform. Thus, Khare et al. [26] observed a decrease in microbiome biodiversity and short-chain fatty acid production in rats fed a diet containing wood-derived cellulose nanofibers. Lopes et al. [30], in an in vitro system, observed suppression of *Escherichia coli* growth in the presence of plant cellulose nanofibers with minimal impact on the growth of lactobacilli. At the same time, as follows from the most recent systematic review [31], the microbiological effects of BNC are not described in the literature, and our data on its effect is apparently obtained for the first time. From the results received, it follows that the most pronounced effect of BNC, judging by the number of affected microbial populations, is observed at an intermediate (10 mg/kg) dose of this nanomaterial. The non-monotonic nature of the dose–response relationship for BNC was shown in our previous work for the behavioral indicators of rats in the elevated plus maze (EPM) test [11]. The absence of a clear relationship between the consumed dose and the response of a biological object is a fairly common situation in nanotoxicological studies, which apparently is associated with the achievement of a maximum effective dose of free (non-aggregated) nano-objects in the biological environment (in this case, short fragments of cellulose nanofibrils in the intestinal contents) at an intermediate concentration of nanomaterial, above which this effective dose tends to decrease due to the prevailing aggregation processes according to the law of mass action [32]. The effective dose of nanomaterials should generally be determined not on the basis of mass concentration but on the number of individual particles. For nanofiber materials, as the mass concentration decreases, the number of individual particles (short fiber fragments) that can penetrate biological barriers and be captured by living cells may increase. In the case of nanocellulose, a “bell-shaped” dose–response relationship was observed, for example, when assessing nephrotoxicity endpoints in a study [33]. In previous studies [16,17], which showed the absence of acute and subacute toxicity in BNC, it was administered to animals in quantities that were more than two orders of magnitude greater than those practically achieved when BNC was used as a food additive, which, apparently, made it impossible to detect any effects, which can manifest at significantly lower effective doses. Determining the exposure dose of individual BNC fibers in condensed media, especially in a biological environment, presents a number of methodological challenges and uncertainties.

Our data have shown that the influence of BNC on the composition of intestinal microbiome populations was ambiguous. If at an intermediate dose of 10 mg/kg b.w. both potentially undesirable (overproduction of aerobic hemolytic microflora) and favorable (suppression of the development of enterococci, stimulation of lactobacilli growth) changes are observed, then at the highest dose of BNC (100 mg/kg), the number of molds significantly decreases, which should also be seen as a positive factor. Thus, the data obtained do not exclude the presence of prebiotic effects in BNC, which should be the subject of more detailed studies.

One of the possible consequences of consuming fibrous nanomaterials, such as BNC, may be their effect on the state of the protective mucosal barrier of the intestine, which is initially due to the presence of the so-called “mucoadhesive action”, that is, the ability to bind to the layer of intestinal mucus and influence its rheological properties and permeability to antigens and bacterial toxins [9]. Secondary to this may be an effect on the expression of proteins that ensure the consistency of intercellular contacts of the intestinal epithelium (claudins, occludins, caveolins, cadherins, etc.), an increase in the macromolecular permeability of the intestinal wall for microbial toxins, and increased allergic sensitization [9,26]. Despite this, the BNC, along with its fragments, is apparently not bioavailable on its own. These results were obtained in a study where BNC, when consumed daily for 21 days by Wistar rats, was visualized using fluorescence microscopy only in the intestinal lumen [34]. In a study [35], no signs of toxic and pro-oxidant effects of plant nanofibrous cellulose on the intestinal epithelium of rats and the Caco_2_ monolayer of human intestinal epithelial cells could be identified. However, it is difficult to compare the results of studies presented in the literature on this issue due to differences in the properties of the nanocellulose used and the characteristics of the experimental models. In our work, a significant effect of BNC on the state of the mucosal barrier, monitored by the level of the iFABP2 protein, was also not observed, at least at medium and high doses of the nanomaterial when the levels of this biomarker remained mainly within normal values. At the same time, an abnormal increase in iFABP2 concentration was observed in some animals at the lowest dose of 1 mg/kg. At the same dose of BNC, the greatest degree of desquamation of enterocytes of the villi of the small intestinal mucosa was observed. The decrease in villus length and villus/crypt ratio in BNC-fed rats was consistent with changes in the intestinal epithelium induced by conventional cellulose consumption previously observed in monkeys [36]. However, the lack of dependence of these effects on the dose of nanomaterial does not allow them to be correctly taken into account when assessing the NOEL.

## 5. Conclusions

Thus, this study demonstrated that when administered to rats with a diet for 8 weeks, BNC at a dose of 100 mg/kg b.w. exerts potentially unfavorable changes in hematological and immunological blood parameters, lipid metabolism parameters, and liver tissue morphology, indicating the development of chronic systemic inflammation. These changes do not directly correlate with behavioral reactions (level of anxiety and cognitive function, see [11]), with shifts in the intestinal microbiome and possible effects on the morpho-functional state of the mucosal barrier of the small intestine, and also do not lead to increased allergic sensitivity in the animals. At a BNC dose of 100 mg/kg b.w. we identified vacuolization of hepatocytes in the periportal region of the liver parenchyma and minimal disturbances in lipid and nitrogen metabolism [11]. The mechanism of development of these changes is not clear enough and may presumably be associated with the presence of unidentified forms of micronutrient deficiency caused by BNC in a high dose due to its high adsorptive and ion exchange capacity. Based on the data obtained, it can be tentatively concluded that the maximum non-effective dose of BNC during subacute oral administration is less than 100 mg/kg body weight, which is apparently higher than previously obtained estimates of 5.2–5.3 mg/kg b.w. per day [18]. The studies carried out in this and previous [11] works were aimed at establishing the permissible daily dose of BNC and the possibility of its use as a food ingredient. However, during this experiment, the question arose about the presence of hidden types of micronutrient deficiency in animals, presumably caused by BNC binding with certain nutrients in the intestinal lumen. Further clarification of this circumstance should serve to establish optimal ways to use BNC as a component of foods. In order to accomplish this, it will be essential to conduct separate, long-term studies using modern analytical techniques to assess micronutrient and trace element deficiencies, as well as to cross-validate the animal models employed.

## Data Availability

Data available on request due to restrictions e.g., privacy or ethical.

## Supplementary Materials

The following supporting information can be downloaded at: https://www.mdpi.com/article/10.3390/nano14201678/s1, Table S1: Serum cytokines levels in rats in 8-week experiment of BNC feeding.
